# Motion artifact correction for resting-state neonatal functional near-infrared spectroscopy through adaptive estimation of physiological oscillation denoising

**DOI:** 10.1117/1.NPh.9.4.045002

**Published:** 2022-10-22

**Authors:** Mingxi Yang, Meiyun Xia, Shen Zhang, Di Wu, Deyu Li, Xinlin Hou, Daifa Wang

**Affiliations:** a Beihang University, Ministry of Education, School of Biological Science and Medical Engineering, Beijing Advanced Innovation Center for Biomedical Engineering, Key Laboratory of Biomechanics and Mechanobiology, Beijing, China; b Beihang University, School of Mechanical Engineering and Automation, State Key Laboratory of Virtual Reality Technology and System, Beijing, China; c Peking University First Hospital, Department of Neonatal Ward, Beijing, China

**Keywords:** functional near-infrared spectroscopy, neonatal resting-state data, motion artifacts correction

## Abstract

**Significance:**

Functional near-infrared spectroscopy (fNIRS) for resting-state neonatal brain function evaluation provides assistance for pediatricians in diagnosis and monitoring treatment outcomes. Artifact contamination is an important challenge in the application of fNIRS in the neonatal population.

**Aim:**

Our study aims to develop a correction algorithm that can effectively remove different types of artifacts from neonatal data.

**Approach:**

In the study, we estimate the recognition threshold based on the amplitude characteristics of the signal and artifacts. After artifact recognition, Spline and Gaussian replacements are used separately to correct the artifacts. Various correction method recovery effects on simulated artifact and actual neonatal data are compared using the Pearson correlation (R) and root mean square error (*RMSE*). Simulated data connectivity recovery is used to compare various method performances.

**Results:**

The neonatal resting-state data corrected by our method showed better agreement with results by visual recognition and correction, and significant improvements (R=0.732±0.155, RMSE=0.536±0.339; paired t-test, **p<0.01). Moreover, the method showed a higher degree of recovery of connectivity in simulated data.

**Conclusions:**

The proposed algorithm corrects artifacts such as baseline shifts, spikes, and serial disturbances in neonatal fNIRS data quickly and more effectively. It can be used for preprocessing in clinical applications of neonatal fNIRS brain function detection.

## 1 Introduction

Functional near-infrared spectroscopy (fNIRS) is an emerging neuroimaging method.^1^^–^^3^ By emitting specific wavelengths of near-infrared light (650 to 900 nm) toward the scalp and measuring the intensity of the scattered light, the relative changes in oxyhemoglobin (HbO) and deoxyhemoglobin (HbR) in the cerebral cortex can be obtained, which provides information on the brain activity and the relationship between the various brain regions.^4^^,^^5^ Owing to its non-radiative, noninvasive, and patient-friendly nature, this technology is widely used for the detection of brain function in autism,^6^ hyperactivity disorder,^7^^,^^8^ and infants.^9^^–^^11^ Especially in the case of newborns, fNIRS is more robust and suited to the free movements of the newborn than other neuroimaging techniques.^12^^,^^13^ As a useful supplement to existing techniques, fNIRS can provide clinicians with information on functional brain imaging and gain insight into cerebral development^14^ and mechanisms of injury in neonates by monitoring the brain function of neonates and preterm infants^15^^–^^17^ with brain injury.^18^^–^^20^

One challenge that remains when using fNIRS, a promising brain imaging method, for rapid examination of neonatal populations is the interference of motion artifacts.^13^^,^^18^ During data acquisition, the relative motion between the light source, detector, and neonatal scalp^21^^,^^22^ leads to interference in the fNIRS signal with motion artifacts,^23^ such as spikes, baseline shifts, or serial disturbances^24^ consisting of a mixture of spikes and baseline shifts for a few seconds. There are two main reasons for this phenomenon. First, newborns cannot follow instructions as adults can.^25^^,^^26^ Even during sleep after full lactation, they appear to move their heads randomly.^13^^,^^20^ Second, a neonatal skull is soft, the fontanelle is not closed, and other tissues around the brain are relatively thin. To prevent the detector and light source from causing damage or pain to the newborn, the light source and detector should be gently applied to the neonate’s head, and no external force is allowed to be applied to the skull during neonatal data acquisition.^20^ Small movements of the newborn can cause slippage between the light source, detector, and scalp. These unavoidable problems can lead to motion artifacts contaminating the fNIRS data.^27^

Typically, motion artifacts in fNIRS data have peaks or offsets much higher than the range of physiological oscillations, leading to discrepancies between the information reflected by the contaminated fNIRS signal and the true activity of the cortex.^27^^–^^29^ Inappropriate preprocessing procedures or uncorrected motion artifacts generally result in false-positive or false-negative results.^18^ Functional connectivity is a prevalent method of analysis, particularly in the study of resting-state data.^19^ Abrupt artifacts generally occur in multiple channels simultaneously, causing false correlations and confounding the conclusions.

Correction algorithms have been proposed in the field of fNIRS to reduce the interference of motion artifacts. A simple method is to discard the artifact segments. The signal is corrected by removing the motion artifacts.^30^ However, this method is not applicable when faced with the difficulty of intercepting long-time artifact-free data segments.^31^ Some researchers have corrected the motion artifacts according to their characteristics or frequencies through methods, such as principal component analysis method (PCA)^32^ and wavelet filtering (WAVE).^33^ PCA first converts the signals into orthogonal principal component sets. The transformed variance, which is relatively large, represents the motion artifacts. The signal is reconstructed after discarding the components that account for a relatively large variance. However, this approach requires multiple channels and tends to overcorrect signals. To avoid this problem, an improved method called target PCA (tPCA)^34^ was developed to correct the data marked as artifacts. The WAVE method first decomposes the fNIRS signal into wavelet coefficients. The wavelet coefficients of the motion artifacts appear as outliers among all the coefficients. The motion artifact is corrected by removing the outliers from all wavelet coefficients and reconstructing the signal. This approach is more effective in correcting spikes and high-frequency noise than baseline shifts of the signals.^33^^,^^35^ An effective method for correcting baseline shifts is the movement artifact reduction algorithm, also known as spline interpolation (Spline).^36^ Spline first distinguishes between artifacts from all signals based on the moving standard deviation (MSD). Motion artifacts are modeled using cubic spline interpolation, and the model is subtracted from the original signal. Finally, the average values before and after the motion artifact segments are adjusted to obtain the denoised signal.^36^^–^^38^ Spike noise may remain in the corrected signal because of the weak ability of Spline to fit the spikes.^36^^,^^39^ Another method to correct the signal using the physiological properties of the variation in hemoglobin concentration is the correlation-based signal improvement method (CBSI).^40^ This method eliminates motion artifacts by assuming that HbO and HbR are always negatively correlated. It is mathematically simple and requires fewer parameters to be set. However, the assumption may not always hold and may have limitations for subjects with abnormal brain activity.^31^^,^^41^

The above methods have been widely used in various applications of fNIRS but have not been adequately studied for neonatal fNIRS data. Behrendt et al. corrected the fNIRS data for three ages (5, 7, and 12 months) of infants using traditional motion correction methods. The data were combined with a simulated hemodynamic response function (HRF). The results showed that the WAVE method at specific coefficients could remove noise from the data and recover the true HRF.^22^ However, because the study used simulated HRF, and the actual HRF of infants usually has small fluctuations^25^ and uncertainty,^42^ the effectiveness of the direct correction of infant data may need to be validated. Jahani et al.^24^ proposed a parameter-independent Spline combined with Savitzky–Golay (SG) filtering. This algorithm performs spline interpolation correction and SG filtering when the signal-to-noise ratio of the data is >3. Only SG filtering is applied when the signal-to-noise ratio is <3. This approach was validated using adult fNIRS data. However, Di Lorenzo et al. pointed out that the signal-to-noise ratio of infant data is generally lower than three. Therefore, only the Golay filter should be used for infant data, which is not sufficient to correct motion artifacts.^27^ Some researchers have explored the effectiveness of different combinations of correction methods in artifact removal. Di Lorenzo et al.^27^ used the fNIRS data collected from infants aged 4 to 11 months while performing different tasks to explore the effectiveness of the correction methods. They also combined the simulated hemodynamic functions. Their results showed that the corrected results obtained with the combined Spline and WAVE approach (Spline-WAVE) were closer to the actual HRF. Further, it was pointed out that the performance of the method was highly dependent on the detection of motion artifacts.^27^ Motion artifacts are automatically detected by observing whether the MSD of the data within the sliding window exceed the magnification of the standard deviation (STD).^36^ However, direct use of neonatal data with a large number of artifacts can cause STD values to be high, resulting in missing motion artifacts. In summary, traditional motion artifact correction methods have certain limitations in the processing of neonatal fNIRS data. These problems can cause neonatal fNIRS signal preprocessing results that may not be consistent with the actual situation. Thus, handling neonatal data with dense motion artifacts is challenging for researchers and pediatricians.

Facing the above challenges, we consider that the identification of motion artifacts should be based on objective detection of physiological oscillation changes in each channel. In addition, different types of noise should be removed separately to avoid residuals. Based on this idea, we designed a motion artifact correction method in this study to remove different types of artifacts from resting-state neonatal fNIRS data. The method first estimates the STD of the physiological oscillations in each channel of the signal, which is then used as a threshold to mark the motion artifacts accurately. Then, the baseline shift and spike noise are corrected separately using the respective advantages of Spline and Gaussian replacement. In addition, other correction methods, Spline, WAVE, CBSI, tPCA, Spline-SG, and Spline-WAVE were used in this study. Neonatal resting-state data and simulated artifact data were used together to validate the effectiveness of different artifact correction methods. We first compared the detection efficiency of our method with the traditional methods for detecting motion artifacts. Then, after applying each motion correction algorithm, the change in HbO after correction by the different algorithms was compared with the established criteria in terms of the following metrics: root mean square error (*RMSE*) and Pearson’s correlation (R). The criterion for the neonatal data was the change in concentration of HbO visually identified and corrected, while the criterion for the simulation data was the change in HbO concentration when no artifacts were added. In addition, connectivity changes were used to assess the performance of the algorithm. The connectivity of homologous channels in the simulation data and the connectivity between different regions were used as our metrics of interest.

## 2 Method

### 2.1 Adaptive Estimation of Physiological Oscillation Denoising

**Fig. 1:**
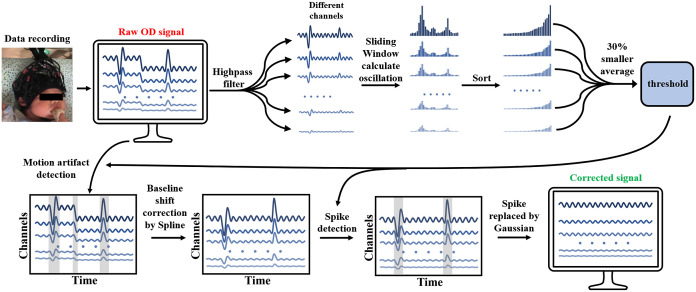
AEPO workflow, including estimation of physiological oscillations, artifact identification, and correction.

Motion artifacts in the fNIRS signals are noises with short time variations and large amplitudes. A suitable threshold value is required to accurately identify the motion artifacts. In addition, to avoid residual artifacts, different types of artifacts must be corrected separately in different ways. To address the above issues, this study first identified motion artifacts in the fNIRS signals based on their characteristics and obtained the deviation of the noise-free signal portion of each channel using an estimation method, which was used as the threshold to identify the artifacts. Finally, the artifacts were corrected using Spline and Gaussian white noise replacement. The method is shown in Fig. 1.

#### 2.1.1 Estimated physiological oscillations

In the neonatal fNIRS data, the frequency, amplitude, and time of occurrence of motion artifacts are random. The basis of detecting motion artifacts is the determination of whether the MSD of the data within the sliding time window exceeds the threshold by a certain multiple. In previous studies, this threshold reference value has been used as the STD of the overall data difference. For widely varying neonatal data, even a careful adjustment of the multiple will not always satisfy this requirement.

Generally, the motion artifacts in fNIRS signals occupy a portion of the entire signal. If the fNIRS data are divided into small segments, the segments should include both noiseless data and data with artifacts. Therefore, we calculated the STD of the physiological oscillations for all small-segment data. In this set of STDs, smaller values correspond to the noise-free parts of the signal. Therefore, the smaller values were averaged to obtain the deviation of the overall data as the threshold. In the previous method,^36^ the STD of the entire data difference was used as the threshold. However, the STD becomes uncertain because of the amplitude and frequency of the artifacts in the signal. The improvements proposed in our study are that the threshold is related only to the physiological oscillation part of the signal and is relatively stable.

The procedure of the proposed method is as follows: after obtaining the neonatal fNIRS signal, the original signal is transformed into an optical density (OD) signal and 0- to 0.75-Hz bandpass filtering is performed to remove the effects of the slow drift on STD calculations. The filtered signal is used to estimate the threshold value. Next, the STD of the physiological oscillations for each small fluctuation is calculated using the sliding window method (4 s window with 2 s interval)^43^

$$\mathrm{STD}=\sqrt[{2}]{\frac{1}{N-1}\overset{N-1}{\underset{t=1}{\sum }}{\left[X(t+1)-X(t)-\frac{1}{N-1}(\overset{N-1}{\underset{t=1}{\sum }}X(t+1)-X(t))\right]}^{2}},$$ (1)

where N is the window length and X(t) is the OD data. Next, the STDs are arranged in ascending order. The STDs of the artifact-free part are more concentrated in the first 1/3 interval of the entire set. Therefore, the top 30% are extracted and averaged as the STD of the noise-free part of the signal.

#### 2.1.2 Motion artifact recognition

After estimating the STD of the physiological oscillations of the signal, its product with the threshold multiplier is used as the recognition threshold. This threshold multiplier is consistent with STDEVthresh used in Homer2, and we set this parameter to 7.^39^ Next, we determine whether the difference between two points before and after the signal exceeds the threshold value of the sliding window. If it exceeds, it is marked as a motion artifact. In this study, a modified hmrMotionArtifactbyChannel (hMAC) function was used to detect the motion-contaminated part of the signal at the designed threshold.^39^ At the end of the detection step, the entire signal is represented as follows:

$$X(t)=\{X_{\mathrm{FM},1}(t),X_{\mathrm{MA},1}(t),X_{\mathrm{FM},2}(t),X_{\mathrm{MA},2}(t),\ldots ,X_{\mathrm{FM},L}(t),X_{\mathrm{MA},L}(t)\},$$ (2)

where X(t) denotes the data of all channels, $X_{\mathrm{FM}}$ denotes the free-motion part of the signal, and $X_{\mathrm{MA}}$ denotes the motion artifacts in the signal. L represents the channel.

#### 2.1.3 Motion artifact correction

After the artifacts are identified, we first correct the motion artifacts by means of spline interpolation. The identified motion artifacts are first modeled using cubic spline interpolation. Depending on the parameter p in the specified range [0,1], the degree of spline function is chosen to be used. For example, when p=0, a least-squares linear fit is applied, whereas when p=1, a cubic spline interpolation is performed.^36^ In this study, we used the same parameters as in previous studies (p=0.99) to correct for the identified motion artifacts.^24^ Then, the signal fitted by spline interpolation is subtracted from the original signal to obtain the denoised signal. Finally, the average values before and after the motion artifact segment are adjusted to reconstruct the entire time series. The reconstructed signal is expressed as follows:

$$Y(t)=\{X_{\mathrm{FM},1}(t),X_{\mathrm{SP},1}(t)+a_{1},X(t),X_{\mathrm{SP},2}(t)+a_{2},\cdots ,X_{\mathrm{FM},L}(t),X_{\mathrm{SP},L}(t)+a_{L}\},$$ (3)

where Y(t) is the signal after the first correction, and $X_{\mathrm{SP}}$ is the part of the motion artifacts after the Spline correction; and a is the array of difference between the average values before and after the motion artifacts in different channels.

Because the subtraction of the signal leaves a small amount of high-frequency oscillation, the corrected signal contains spikes. Therefore, the artifacts must be identified again using the process described in Sec. 2.1.2 to ensure that the data previously identified as normal will not be marked again after the Spline. The spike noise usually lasts for a short time, and we use Gaussian replacement to repair it. The identified spikes are replaced by Gaussian white noise of the same amplitude as the physiological oscillations of the signal. The Gaussian white noise is calculated as follows:

$$Y_{GS}(t)=\{STD_{1}\times Wgn(t),STD_{2}\times Wgn(t),\cdots ,STD_{L}\times Wgn(t)\},$$ (4)

where $Y_{\mathrm{GS}}(t)$ denotes the fitted signal. The $STD_{L}$ is the physiological oscillation of each channel in the data. Wgn(t) is the Gaussian white noise with a STD of 1. After the replacement, artifact correction is completed by adjusting the signal segments before and after the spike noise. The final reconstructed signal is

$$Z(t)=\{Y_{\mathrm{FM},1}(t),Y_{\mathrm{GS},1}(t)+b_{1},Y_{\mathrm{FM},2}(t),Y_{\mathrm{GS},2}(t)+b_{2},\cdots ,Y_{\mathrm{FM},L}(t),Y_{\mathrm{GS},L}(t)+b_{L}\},$$ (5)

where Z(t) represents the signal after the correction process and $Y_{\mathrm{FM}}$ is the signal without spikes; and b is the array of difference between the average values before and after the spike correction in different channels.

### 2.2 Traditional Artifact Correction Techniques

To verify the effectiveness of the proposed method, we compared it with Spine, WAVE, tPCA, CBSI, Spline-SG, and Spline-WAVE.

The Spline method was proposed by Scholkmann et al. The motion artifact of each channel is first detected.^36^^,^^37^ The corrected signal is the difference between the original signal and the cubic spline interpolation of the artifacts. Then, the entire dataset is reconstructed by moving the average values before and after the artifact segments. In this study, we identified the motion artifacts using the settings *AMPThresh* = 0.5, *TMotion* = 0.5 s, *TMASK* = 1 s, and *STDEVthresh* = 15, 10, 7, 5. According to previous reports, the parameter p was set to 0.99 to correct the identified motion artifact.^24^

Spline-SG is a motion artifact correction method proposed by Jahani et al.^24^ This method uses Spline to correct for baseline drift when the SNR of the signal is >3, and later uses SG filtering to correct for spike artifacts in the signal. If the SNR of the signal is <3, only SG filtering is used for correction. In this study, we set the parameter of SG filtering to 6 s, according to the suggestion of Jahani et al.

Molavi and Dumont^33^ proposed a motion artifact removal method based on discrete wavelet transform. First, the signal is decomposed into wavelet coefficients at different levels. The wavelet coefficients of the motion artifacts are shown as outliers in the fNIRS signal background. Then, the motion artifacts are removed by detecting the outliers in the different wavelet coefficients and setting them to zero. In this study, the interquartile range (*IQR*) was set to 1.5 to detect the outliers after wavelet decomposition for artifact correction.^24^ In addition, we used Spline-WAVE to correct for artifacts. We used IQR of 0.8 in this study by referring to a previous neonatal study.^27^

Zhang et al.^32^ proposed a PCA method in 2005. Yücel et al.^34^ proposed an improved PCA method, called tPCA, based on the above approach for motion artifact removal. This method first arranges the data of all channels into a matrix and then extracts the eigenvalues and eigenvectors. Larger eigenvalues are assumed to represent the differences caused by the motion artifacts, which were removed by removing the feature values. In this study, the algorithm had the same parameter settings as applied in Spline to identify the artifacts: *STDEVthresh* = 15, 10, 7, 5; *AMPThresh* = 0.5; *TMotion* = 0.5 s; *TMASK* = 1 s; and *nSV* = 0.9. The denoised signal was reconstructed after removing 97% of the variance in the motion detection part of the signal. This process was repeated two more times to detect any remaining motion artifacts.

Cui et al. proposed the CBSI method in 2010. The main assumption in this method is that the measured fNIRS signal consists of three components: signal for the change in the true Hb component content, motion artifacts, and white noise. Motion artifacts attenuate the negative correlation between the HbO and HbR concentrations. Thus, the actual HbO and HbR concentrations can be derived from the mathematical relationships established using the previously mentioned assumptions.^40^ The hmrMotionCorrectCbsi function was used for artifact correction in this study.

### 2.3 fNIRS Data

#### 2.3.1 Simulated motion artifact in fNIRS data

This study first used simulated motion artifact data to verify the effectiveness of the proposed method. By referring to previous studies,^38^ we simulated the artifact data by adding artifacts randomly to the noise-free fNIRS signal.

**Fig. 2:**
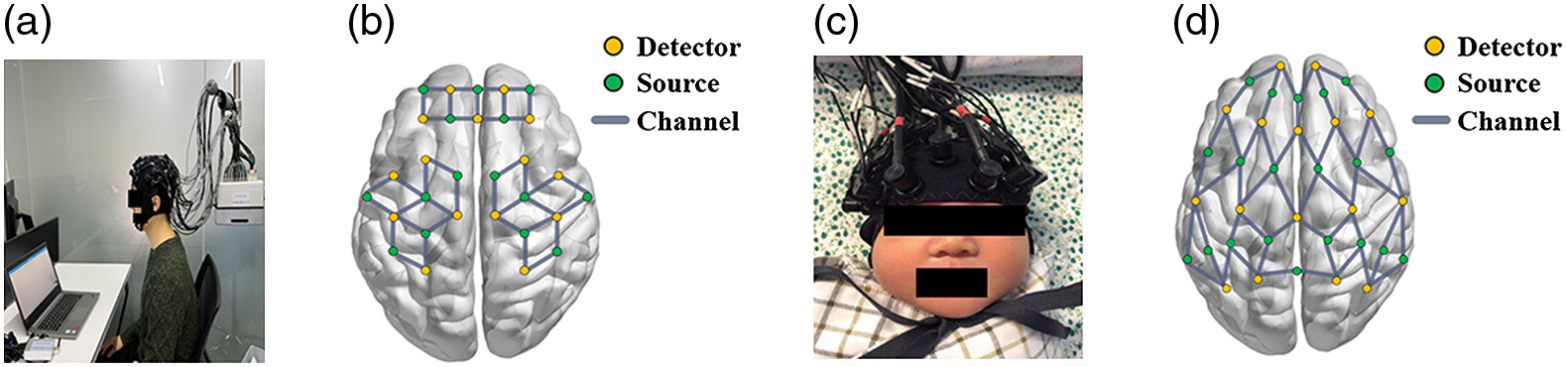
(a) Experimental setting for simulated fNIRS data; (b) fNIRS layout in the simulated data; (c) experimental setting for neonatal fNIRS data; and (d) fNIRS layout in the neonatal signal.

The study recruited one healthy adult subject and recorded the resting-state brain fNIRS signals twice, as shown in Fig. 2(a). The duration of resting-state data acquisition was 8 min. During the first data acquisition, the subject was asked to remain stationary. The second time, the subject was asked to deliberately mimic the movements of the newborn during wakefulness, including head shaking, blinking, and other actions. Dual wavelength (760 and 850 nm) fNIRS signals were acquired using a multichannel fNIRS system (NirSmart, Danyang Huichuang, China). The sampling frequency was 10 Hz. The locations of the monitored brain regions are shown in Fig. 2(b), including the prefrontal and parietal lobes.

Artifacts in the second fNIRS data were identified using hMAC (*STDEVthresh* = 10, *AMPThresh* = 0.5, *TMotion* = 0.5 s, and *TMASK* = 1 s), and then all the artifacts were extracted and formed into an artifact set. About 30 artifact segments were randomly added to the artifact-free data, that included baseline shifts, spikes and serial disturbances. The motion artifact duration exceeded 20% of the overall duration. This operation was repeated 30 times, resulting in 30 simulated artifacts of fNIRS data.

#### 2.3.2 Neonatal fNIRS data

In this study, resting-state neonatal data were collected to explore the effect of our proposed method on different types and degrees of noise in the neonatal fNIRS data. The participants in the experiment were neonates from the Department of Pediatrics, Peking University First Hospital. The inclusion criteria were as follows: (1) full-term newborns, gestational age from 37 to 44 weeks and (2) consent from legal guardians. All relevant assessments were performed within 72 h of the infant’s birth. About 12 newborns were enrolled in the study, including six healthy neonates and six newborns with hypoxic-ischemic brain damage. The socio-demographic information for this study is shown in Table 1. This study was conducted in accordance with the Declaration of Helsinki and approved by the local ethics committee of Beihang University.

**Table 1:** Sociodemographic information for this study.

Neonatal no.	1	2	3	4	5	6
**Healthy**						
GA (week)	38	37	39	40	35	38
Gender	M	M	M	F	F	M
Birth weight (g)	2450	4000	3350	3340	2160	3690
Feeding	Mix	Mix	Mix	Mix	Milk	Milk
**HIBD**						
GA (week)	41	39	40	40	40	37
Gender	F	F	M	F	F	M
Birth weight (g)	3050	3305	2600	3350	3240	2890
Feeding	Mix	Milk	Mix	Milk	Milk	Milk

GA = gestational age; M = male; F = female; mix = formula milk powder + breast feeding; HIBD = hypoxic-ischemic brain damage.

Neonatal data were acquired using a multichannel, dual wavelength (760 and 850 nm) fNIRS system (NirSmart, DanYang HuiChuang, China) at a sampling rate of 10 Hz. A room with appropriate dim lighting and sound insulation was selected to reduce the interference of the external environment on the infants. Before the experiment, the newborn was placed in a supine position in the crib. Owing to the softness of the infant’s skull, light sources and detectors were gently attached to the newborn’s head for safety and comfort, as shown in Fig. 2(c). The fNIRS head cap comprised 20 light sources and 16 detectors. The covered areas were the prefrontal, temporal, and parietal lobes, as shown in Fig. 2(d). The 10-min resting-state data were collected as the neonatal data used in the experiments. All newborns were monitored after full lactation and natural sleep.

### 2.4 Criteria and Indicators for Evaluation

#### 2.4.1 Evaluation criteria

First, the efficiency of adaptive estimation of physiological oscillation (AEPO) and hMAC for the recognition of motion artifacts were compared. For the simulation data, the total length of artifacts added to each simulation data was normalized and set to 100%. For the real neonatal data, the total duration of artifacts recognized visually was normalized and set to 100%. The ratio of the total artifact length recognized by different detection methods to the actual total artifact length was taken as the detection efficiency. Exceeding 100% is over-recognition.

Next, the changes in the HbO concentration (ΔHbO) after correction was compared using different methods because resting-state fNIRS data were used. At the same time, manual correction is used as a complement to the artifact correction method. The comparison criterion for the simulated artifact data was ΔHbO calculated without adding motion artifacts. For the neonatal fNIRS data, manual correction (correction motion^44^ artifacts after visual recognition^45^^–^^47^) were used to remove artifacts. fNIRS researchers with expertise in biomedical engineering and signal processing collaborated with pediatricians to review the resting-state fNIRS data of the neonates and marker-correct the artifacts in the signal. By amplifying the signal in the visual interface, the physiological oscillation waveform was observed. Motion artifacts (baseline drift, spikes, and serial disturbances) that did not match the amplitude and frequency characteristics of the waveform in the channel were flagged. The time of marking was from 0.5 s before to 0.5 s after the appearance of the artifact. For the fNIRS data, pediatricians and researchers examined all channels for artifacts. An automatic correction of the labeled artifacts was then performed. The automatic correction method used a random signal to replace motion artifacts, which is consistent with the method used in nirsLAB.^44^ The value of ΔHbO obtained after visual recognition and correction was used as the standard for comparison with other artifact correction methods.

Finally, the effect on connectivity before and after the motion artifact correction in the simulation data was evaluated. For connectivity, the connectivity of contaminated channels to homologous channels or other brain regions was the metric. Specifically, homologous channel connectivity is the mean value of the correlation coefficient between the contaminated channel and other channels of the same light source over the entire 8-min recording duration. The connectivity of other brain regions was the mean of the correlation coefficients between the channels in the four brain regions and the contaminated channels over the entire 8-min recording duration. Thus, for each artifact correction method, 30 sets of five connectivity values were obtained. The connectivity criterion was the connectivity before adding motion artifacts (five values of fixed connectivity).

#### 2.4.2 Evaluation indicators

We used the following two metrics to compare the standard and corrected ΔHbO obtained with each motion correction algorithm:^48^^,^^49^ (1) the Pearson coefficient (R) and (2) *RMSE* defined as follows:

$$\mathrm{RMSE}=\sqrt[{2}]{\frac{1}{N}\overset{N}{\underset{i=1}{\sum }}[a(t_{i})-b(t_{i}){]}^{2}}.$$ (6)

For the simulated data, $a(t_{i})$ is ΔHbO without the addition of motion signals. For neonatal data, $a(t_{i})$ is ΔHbO after manual correction. $b(t_{i})$ is the value of ΔHbO obtained after processing the data with the different artifact correction methods. R was used to evaluate the similarity between the corrected values obtained with the different processing methods and the established standard. R was close to 1, indicating that the processed data had higher similarity. A smaller RMSE indicates that the result of the artifact correction method does not differ widely from the standard result. Finally, paired t-tests were used to evaluate statistically significant differences between different motion artifacts correction methods and corrected for multiple comparisons using false discovery rate. For connectivity, one-way analysis of variance (ANOVA) with false discovery rate (FDR) correction was used to compare the differences in connectivity between the different methods.

## 3 Results

### 3.1 Motion Correction Performance in Simulation Datasets

In the simulation data, the detection efficiency of hMAC is improved when STDEVthresh becomes smaller. However, when using a strict parameter multiplicity, over-recognition occurs. Table 2 lists the recognition efficiency with different STDEVthresh. In this study, we used STDEVthresh = 7 as the correction parameter for Spline, tPCA, and Spline-WAVE.

**Table 2:** Detection efficiency of different methods in simulation data.

Methods	hMAC-15	hMAC-10	hMAC-7	hMAC-5	AEPO
**Detection efficiency**	22.3% ± 5.0%	50.2% ± 13.6%	68.6% ± 17.0%	123% ± 21.8%	86.9% ± 9.0%

hMAC = hmrMotionArtifactbyChannel.

**Fig. 3:**
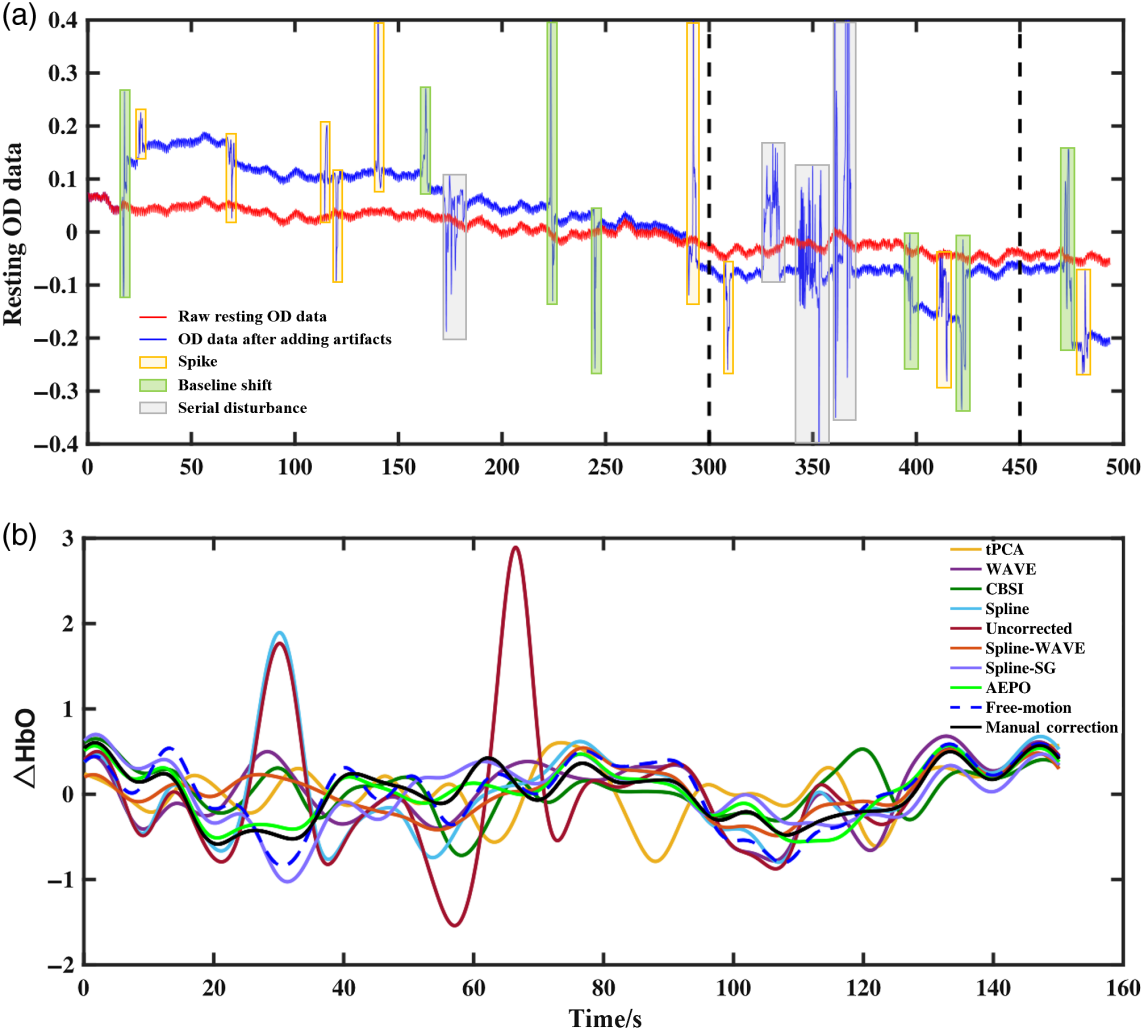
(a) Example of simulated artifact data contaminated by different types of motion: high-frequency spikes (yellow), baseline shifts (green), serial disturbances (gray) and (b) comparison of ΔHbO obtained by different correction methods.

An example of the simulated artifact data used in this study is shown in Fig. 3(a), where the signal with no artifact added is marked in red, and the simulated artifact data are blue. It can be seen from the figure that the simulation data contain baseline shifts, spike artifacts, and serial disturbances, which are identified by boxes of different colors. The entire simulation data accounted for between 25% and 35% of the artifact time. Figure 3(b) shows the effect of ΔHbO after correction using different algorithms, and the raw OD data are boxed in Fig. 3(a). The tPCA and CBSI methods resulted in large deviations of ΔHbO from the true change in HbO concentration, whereas the values of the restored ΔHbO after AEPO, WAVE, Spline-WAVE, and Spline-SG were consistent with the change trend of the true HbO concentration.

**Fig. 4:**
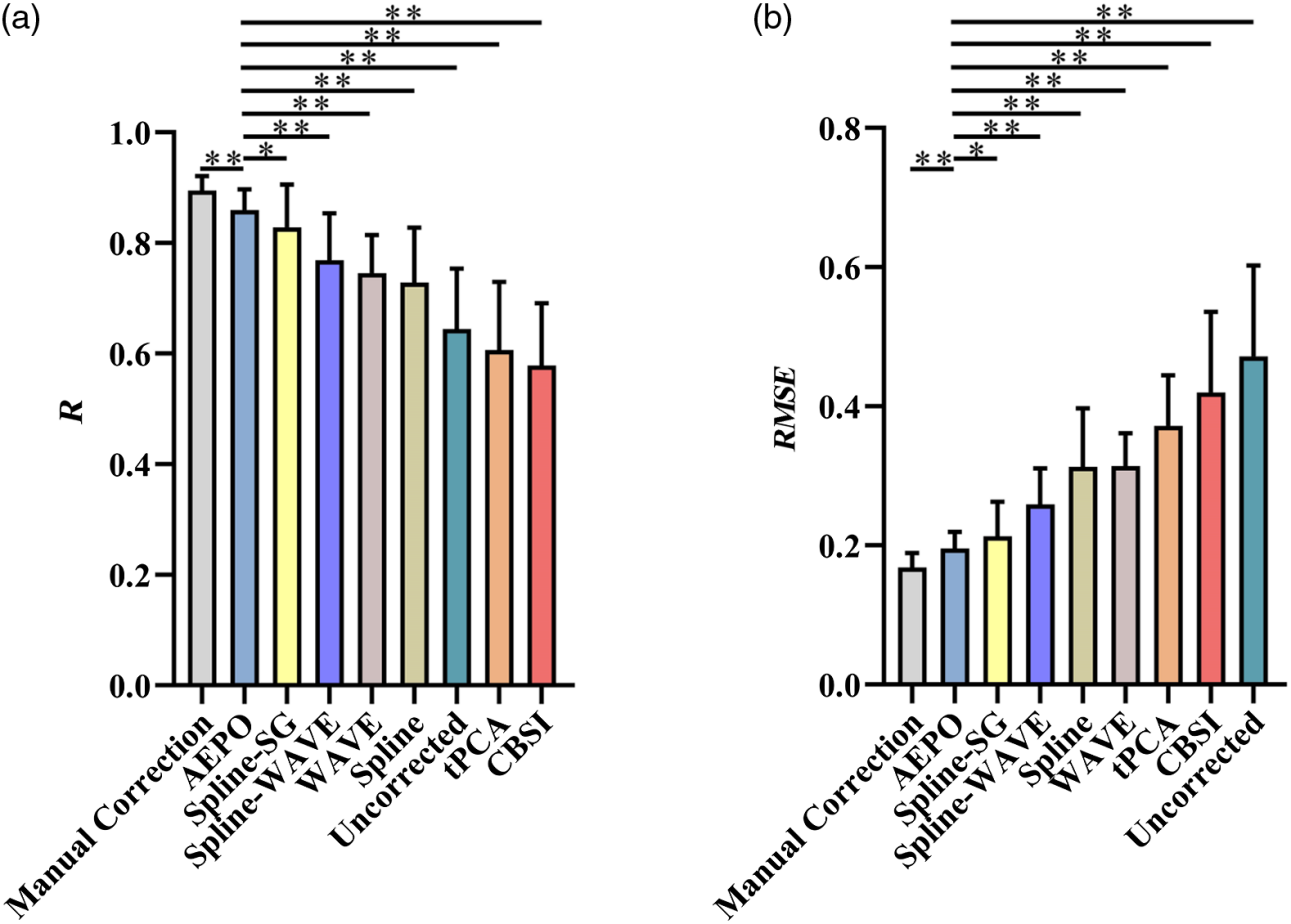
(a) R and (b) *RMSE* values for ΔHbO before (without adding artifacts) and after corrected by different algorithms for the simulation data.

Overall, for the 30 sets of simulated artifact data, the results of the correction methods manual correction (R=0.895±0.025, RMSE=0.168±0.020), AEPO (R=0.860±0.037, RMSE=0.196±0.024), WAVE (R=0.746±0.069, RMSE=0.314±0.048), Spline (R=0.726±0.098, RMSE=0.315±0.084), Spline-WAVE (R=0.767±0.084, RMSE=0.260±0.052), and Spline-SG (R=0.828±0.078, RMSE=0.213±0.049) showed improvements when compared with the uncorrected (R=0.645±0.109, RMSE=0.472±0.131) results. In contrast, tPCA (R=0.613±0.117, RMSE=0.366±0.065) and CBSI (R=0.578±0.113, RMSE=0.420±0.116) did not show improvements, and the difference between the corrected HbO and original HbO changes was large (Table 3 and Fig. 4). Compared with the conventional artifact correction method, the corrected signal obtained with AEPO showed more significant improvements in the two indicators of R and RMSE (paired t-test, **p<0.01, Table 4).

**Table 3:** R and *RMSE* values for simulation data corrected using different algorithms.

Method	R	*RMSE*	Processing time (s)
**Uncorrected**	0.645 ± 0.109	0.472 ± 0.131	0.027
**Manual correction**	0.895 ± 0.025	0.168 ± 0.020	—
**AEPO**	0.860 ± 0.037	0.196 ± 0.024	0.504
**Spline**	0.726 ± 0.098	0.315 ± 0.084	0.145
**WAVE**	0.746 ± 0.069	0.314 ± 0.048	77.844
**tPCA**	0.613 ± 0.117	0.366 ± 0.065	3.830
**CBSI**	0.578 ± 0.113	0.420 ± 0.116	0.077
**Spline-WAVE**	0.767 ± 0.084	0.260 ± 0.052	78.406
**Spline-SG**	0.828 ± 0.078	0.213 ± 0.049	11.640

**Table 4:** P-values (paired t-tests) signifying improvement or significant improvement in R and *RMSE* for the simulation data, calculated as the method in the row over the method in the column.

R RMSE	AEPO	Spline-SG	Spline-WAVE	Spline	WAVE	tPCA	CBSI	Uncorrected
**Manual correction**	<0.01	<0.01	<0.01	<0.01	<0.01	<0.01	<0.01	<0.01
	<0.01	<0.01	<0.01	<0.01	<0.01	<0.01	<0.01	<0.01
**AEPO**		<0.05	<0.01	<0.01	<0.01	<0.01	<0.01	<0.01
		<0.05	<0.01	<0.01	<0.01	<0.01	<0.01	<0.01
**Spline-SG**			<0.01	<0.01	<0.01	<0.01	<0.01	<0.01
			<0.01	<0.01	<0.01	<0.01	<0.01	<0.01
**Spline-WAVE**				0.12	<0.01	<0.01	<0.01	<0.01
				<0.01	<0.01	<0.01	<0.01	<0.01
**Spline**					0.29	<0.01	<0.01	<0.01
					0.95	<0.01	<0.01	<0.01
**WAVE**						<0.01	<0.01	<0.01
						<0.05	<0.01	<0.01
**tPCA**							0.24	0.26
							0.09	<0.01
**CBSI**								<0.05
								<0.01

**Fig. 5:**
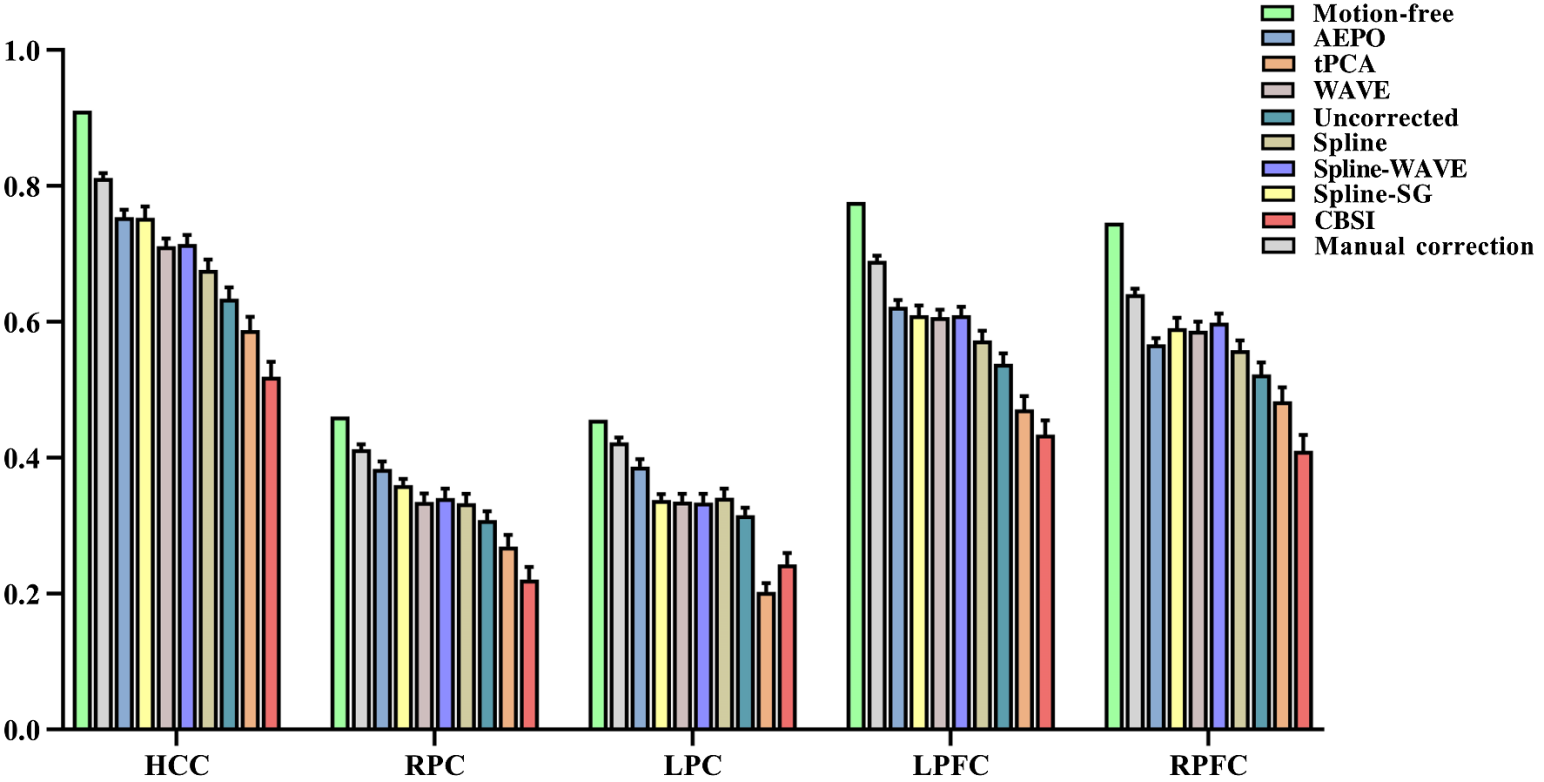
Bar graphs of HCC, RPC, LPC, LPFC, and RPFC in the datasets, including artifact-free data and, after artifact addition, corrected by different methods.

For the analysis of connectivity, the correlation between contaminated channel and the homologous (*HCC*: 0.911), left prefrontal (*LPFC*: 0.777), right prefrontal (*RPFC*: 0.746), left parietal (*LPC*: 0.456), and the right parietal (*RPC*: 0.461) channels before adding motion artifacts were calculated. These indicators decreased after the addition of motion artifacts (*HCC*: 0.633, 0.418 – 0.755; *LPFC*: 0.538, 0.336 – 0.667; *RPFC*: 0.523, 0.304 – 0.665; *LPC*: 0.316, 0.160 – 0.407; *RPC*: 0.308, 0.100 – 0.436). Figure 5 shows the results of connectivity after processing by different methods.

In this study, different methods restored connectivity to different degrees. AEPO increased *HCC* to 0.754 (0.587 – 0.840), *LPFC* to 0.622 (0.510 – 0.724), *RPFC* to 0.567 (0.493 – 0.675), *LPC* to 0.387 (0.298 – 0.503), and *RFC* to 0.384 (0.236 – 0.521). Spline-SG retrieved *HCC* to 0.753 (0.530 – 0.866), *LPFC* to 0.610 (0.406 – 0.715), *RPFC* to 0.591 (0.388 – 0.715), *LPC* to 0.338 (0.232 – 0.423), and *RFC* to 0.360 (0.251 – 0.446). Spline-WAVE increased *HCC* to 0.699 (0.418 – 0.815), *LPFC* to 0.591 (0.402 – 0.723), *RPFC* to 0.551 (0.331 – 0.718), *LPC* to 0.308 (0.147 – 0.422), and *RFC* to 0.323 (0.160 – 0.424). WAVE increased *HCC* to 0.711 (0.530 – 0.795), *LPFC* to 0.607 (0.445 – 0.703), *RPFC* to 0.586 (0.371 – 0.698), *LPC* to 0.335 (0.180 – 0.440), and *RFC* to 0.335 (0.157 – 0.455). The performances recovered by different methods are listed in Table 4.

Table 5 gives the analysis of recovering connectivity by different methods. Compared to uncorrected, tPCA, and CBSI, AEPO showed significant advantages in *HCC*, *LPFC*, *LPC*, and *RPC*. While for Spline, Spline-SG, WAVE, and the AEPO method showed a clear advantage for a part of the connectivity. For Spline-WAVE, AEPO showed no significant difference between the two methods, although it was greater than Spline-WAVE in recovering connectivity. Notably, all connectivity did not return to contamination levels after different motion artifact correction (Table 6).

**Table 5:** *HCC*, *RPC*, *LPC*, *LPFC*, and *RPFC* after the different correction methods.

Method	*HCC*	*RPC*	*LPC*	*LPFC*	*RPFC*
**Motion-free**	0.911	0.461	0.456	0.777	0.746
**Uncorrected**	0.633(0.418 – 0.744)	0.308(0.100 – 0.436)	0.316(0.160 – 0.407)	0.538(0.336 – 0.667)	0.523(0.304 – 0.665)
**Manual correction**	0.812(0.721 – 0.882)	0.413(0.322 – 0.468)	0.422(0.323 – 0.484)	0.690(0.602 – 755)	0.640(0.548 – 0.726)
**AEPO**	0.754(0.578 – 0.840)	0.384(0.236 – 0.521)	0.387(0.298 – 0.503)	0.622(0.510 – 0.724)	0.567(0.493 – 0.675)
**tPCA**	0.588(0.336 – 0.782)	0.269(0.084 – 0.387)	0.203(–0.033 – 0.312)	0.471(0.229 – 0.650)	0.483(0.176 – 0.689)
**WAVE**	0.711(0.530 – 0.795)	0.335(0.157 – 0.455)	0.335(0.180 – 0.440)	0.607(0.455 – 0.703)	0.586(0.371 – 0.698)
**Spline**	0.676(0.482 – 0.824)	0.333(0.148 – 0.447)	0.341(0.182 – 0.445)	0.573(0.426 – 0.707)	0.558(0.391 – 0.683)
**Spline-WAVE**	0.714(0.500 – 0.820)	0.341(0.172 – 0.439)	0.334(0.185 – 0.426)	0.610(0.448 – 0.710)	0.599(0.413 – 0.720)
**Spline-SG**	0.753(0.530 – 0.866)	0.360(0.251 – 0.446)	0.338(0.232 – 0.423)	0.610(0.406 – 0.715)	0.591(0.388 – 0.715)
**CBSI**	0.519(0.323 – 0.740)	0.221(–0.007 – 0.378)	0.243(0.067 – 0.402)	0.434(0.192 – 0.673)	0.410(0.122 – 0.669)

**Table 6:** P-values (one-way ANOVA) signifying improvement in *HCC*, *RPC*, *LPC*, *LPFC*, and *RPFC* for the simulation data corrected by different methods.

Methods	*HCC*	*LPFC*	*RPFC*	*LPC*	*RPC*
MC versus AEPO	<0.01	<0.01	<0.01	0.17	0.41
MC versus tPCA	<0.01	<0.01	<0.01	<0.01	<0.01
MC versus WAVE	<0.01	<0.01	<0.05	<0.01	<0.01
MC versus CBSI	<0.01	<0.01	<0.01	<0.01	<0.01
MC versus Un	<0.01	<0.01	<0.01	<0.01	<0.01
MC versus Spline	<0.01	<0.01	<0.01	<0.01	<0.01
MC versus Sp-Wa	<0.01	<0.01	0.20	<0.01	<0.01
MC versus Sp-SG	>0.05	<0.01	0.13	<0.01	<0.01
AEPO versus tPCA	<0.01	<0.01	<0.05	<0.01	<0.01
AEPO versus WAVE	0.15	0.98	0.95	<0.05	0.09
AEPO versus CBSI	<0.01	<0.01	<0.01	<0.01	<0.01
AEPO versus Un	<0.01	<0.01	0.36	<0.01	<0.01
AEPO versus Spline	<0.01	0.12	>0.99	0.19	0.12
AEPO versus Sp-Wa	0.37	>0.99	0.57	0.07	0.27
AEPO versus Sp-SG	>0.99	>0.99	0.91	<0.05	0.76
tPCA versus WAVE	<0.01	<0.01	<0.01	<0.01	0.07
tPCA versus CBSI	0.35	0.93	0.36	0.60	0.59
tPCA versus Un	0.69	0.17	0.86	<0.01	0.67
tPCA versus Spline	<0.05	<0.01	0.09	<0.01	0.11
tPCA versus Sp-Wa	<0.01	<0.01	<0.01	<0.01	<0.05
tPCA versus Sp-SG	<0.01	<0.01	<0.01	<0.01	<0.01
WAVE versus CBSI	<0.01	<0.01	<0.01	<0.01	<0.01
WAVE versus Un	<0.05	<0.05	0.12	0.94	0.85
WAVE versus Spline	0.68	0.60	0.87	>0.99	>0.99
WAVE versus Sp-Wa	>0.99	>0.99	>0.99	>0.99	>0.99
WAVE versus Sp-SG	0.48	>0.99	>0.99	>0.99	0.80
CBSI versus Un	<0.01	<0.01	<0.01	<0.05	<0.01
CBSI versus Spline	<0.01	<0.01	<0.01	<0.01	<0.01
CBSI versus Sp-Wa	<0.01	<0.01	<0.01	<0.01	<0.01
CBSI versus Sp-SG	<0.01	<0.01	<0.01	<0.01	<0.01
Un versus Spline	0.65	0.75	0.82	0.86	0.93
Un versus Sp-Wa	<0.05	<0.05	0.04	0.98	0.73
Un versus Sp-SG	<0.01	<0.05	0.10	0.80	>0.05
Spline versus Sp-Wa	0.65	0.55	0.51	>0.99	>0.99
Spline versus Sp-SG	<0.05	0.66	0.83	>0.99	0.81
Sp-Wa versus Sp-SG	0.67	>0.99	>0.99	>0.99	0.96

Un = Uncorrected; Sp-Wa = Spline-WAVE; Sp-SG = Spline-SG; MC = Manual correction.

### 3.2 Motion Correction Performance in Neonatal Datasets

About 12 neonatal fNIRS data were collected in this experiment, which included data from six healthy neonates and six neonates with hypoxic-ischemic brain damage. Because the light source could not be close to the scalp during the acquisition of neonatal fNIRS data, we eliminated the channels with no physiological information signal or oversaturated channels from the raw data during data processing (raw light intensity data values >1800 or <0.5). Finally, 409 channels of neonatal fNIRS data were analyzed.

Table 7 lists the detection efficiency of the hMAC with the AEPO method for motion artifacts in the 12 neonatal data. As the STDEVthresh becomes smaller, the detection efficiency increases for some of the data, while over-identification occurs for the others. In this study, for each neonatal data, we selected STDEVthresh with better recognition efficiency without over-recognition as the parameter for Spline, tPCA, and Spline-WAVE.

**Table 7:** Detection efficiency of different methods in newborn data.

Methods	hMAC-15	hMAC-10	hMAC-7	hMAC-5	AEPO
Subject 1	21.4%	35.8%	53.5%	72.0%	89.5%
Subject 2	16.3%	27.7%	41.0%	62.2%	85.3%
Subject 3	24.7%	36.5%	72.6%	142.5%	97.6%
Subject 4	52.6%	65.7%	79.1%	95.2%	98.1%
Subject 5	24.4%	56.0%	93.6%	139.5%	90.1%
Subject 6	40.8%	62.7%	80.8%	102.2%	90.8%
Subject 7	22.2%	30.9%	41.5%	54.4%	83.1%
Subject 8	32.6%	58.3%	85.5%	118.3%	93.4%
Subject 9	71.5%	111.6%	165.5%	234.0%	90.9%
Subject 10	49.7%	71.7%	95.2%	122.8%	93.4%
Subject 11	27.6%	45.0%	64.5%	89.3%	86.7%
Subject 12	61.2%	79.5%	106.0%	133.9%	92.3%

hMAC=hmrMotionArtifactbyChannel.

**Fig. 6:**
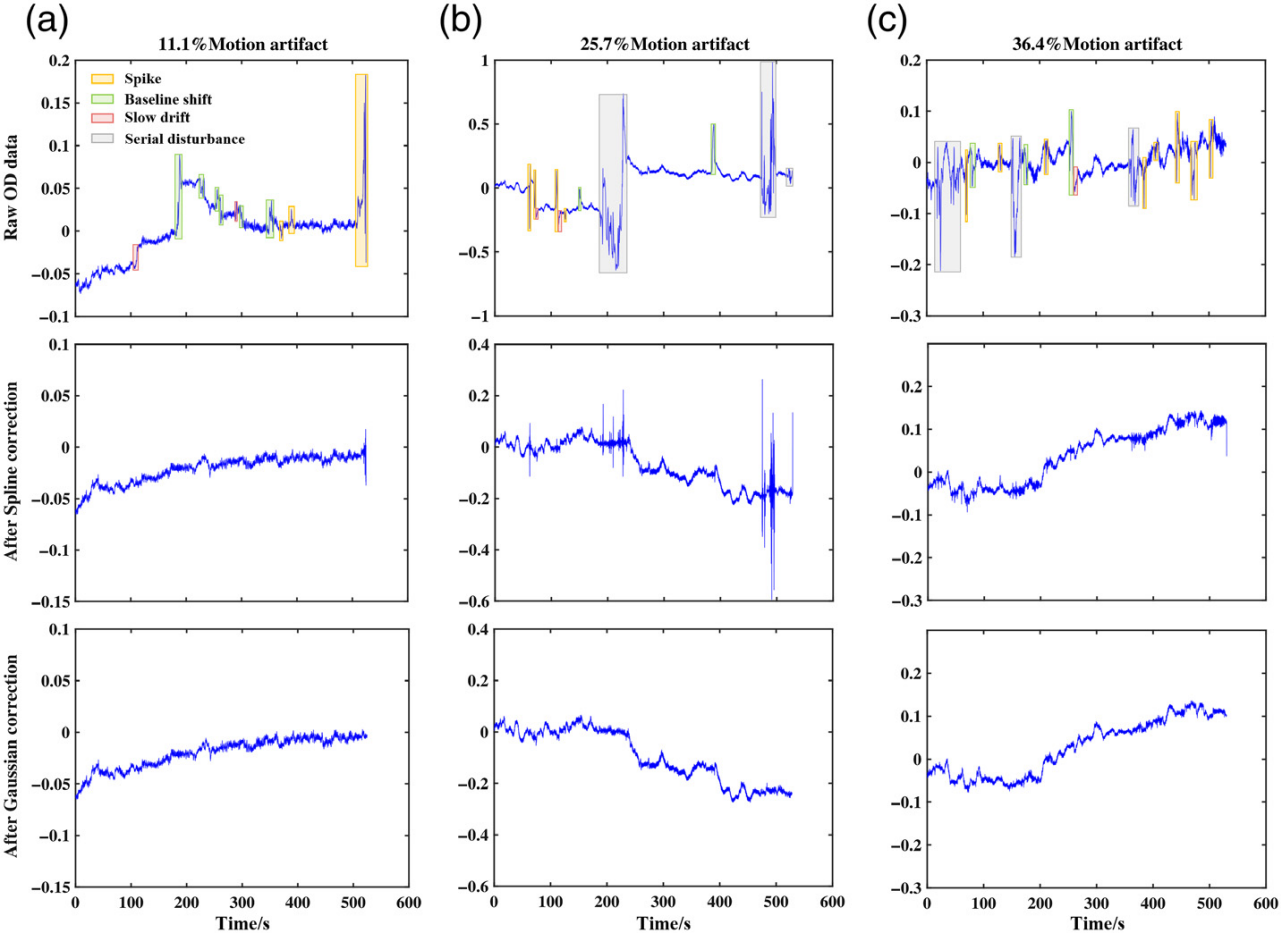
Columns (a)–(c) represent the original neonatal OD signal with low, medium, and high number of artifacts, respectively, and the results after AEPO correction, including that after the Spline and after Gaussian replacement.

Figure 6 shows the correction effect of the neonatal data with different artifact occupancies using the AEPO method. The first row of Fig. 6 represents the raw neonatal OD data containing different numbers of artifacts; in addition to the common baseline shifts, spikes, and slow drift, serial disturbance noise is observed. Different types of noises are highlighted using squares of different colors. The results obtained after the initial detection of artifacts and correction of the signal by applying Spline to the baseline shift is shown in the second row of Fig. 6. After Gaussian replacement to correct the spikes, the final corrected signal is as shown in the third row of Fig. 6.

**Fig. 7:**
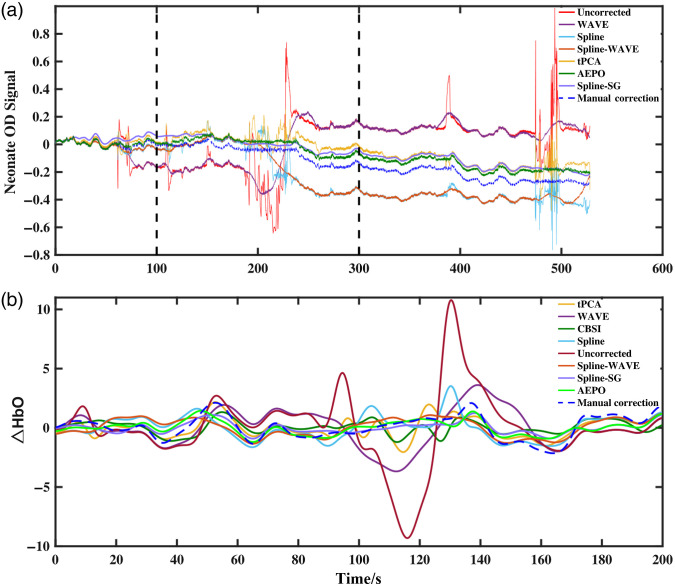
(a) Neonatal OD signal after correction by different methods and (b) ΔHbO after correction with different methods.

To distinguish the results of the proposed method from other correction methods, Fig. 7 shows the OD data after processing with different correction algorithms. Specifically, WAVE removes the spikes in the signal effectively, but it also aggravates the baseline shift of the signal, especially in the parts of the signal containing serial disturbance. In contrast, Spline and tPCA leave residuals when dealing with such perturbations, as shown in Fig. 7(a). The Spline-WAVE method causes baseline shift owing to the residual artifacts. In contrast, the AEPO and Spline-SG methods correct most of the artifacts in the signal. Because the CBSI approach corrects for artifacts based on HbO and HbR, Fig. 7(b) shows ΔHbO after correction using different motion correction techniques, especially in the presence of serial disturbances. As a specific example, the residuals after correction by the Spline, wavelet, and tPCA correction approaches cause a larger deviation of ΔHbO from the manually corrected result (Table 8).

**Table 8:** R and *RMSE* values for neonatal data corrected using different algorithms.

Method	R	*RMSE*	Processing time (s)
**Uncorrected**	0.459 ± 0.227	1.237 ± 0.986	0.055
**AEPO**	0.732 ± 0.155	0.536 ± 0.339	1.258
**Spline**	0.631 ± 0.205	0.700 ± 0.462	0.111
**WAVE**	0.542 ± 0.222	0.811 ± 0.520	100.849
**tPCA**	0.577 ± 0.215	0.666 ± 0.413	0.065
**CBSI**	0.454 ± 0.216	0.777 ± 0.480	0.281
**Spline-WAVE**	0.640 ± 0.191	0.637 ± 0.375	107.539
**Spline-SG**	0.360 ± 0.230	0.966 ± 0.576	23.351

**Fig. 8:**
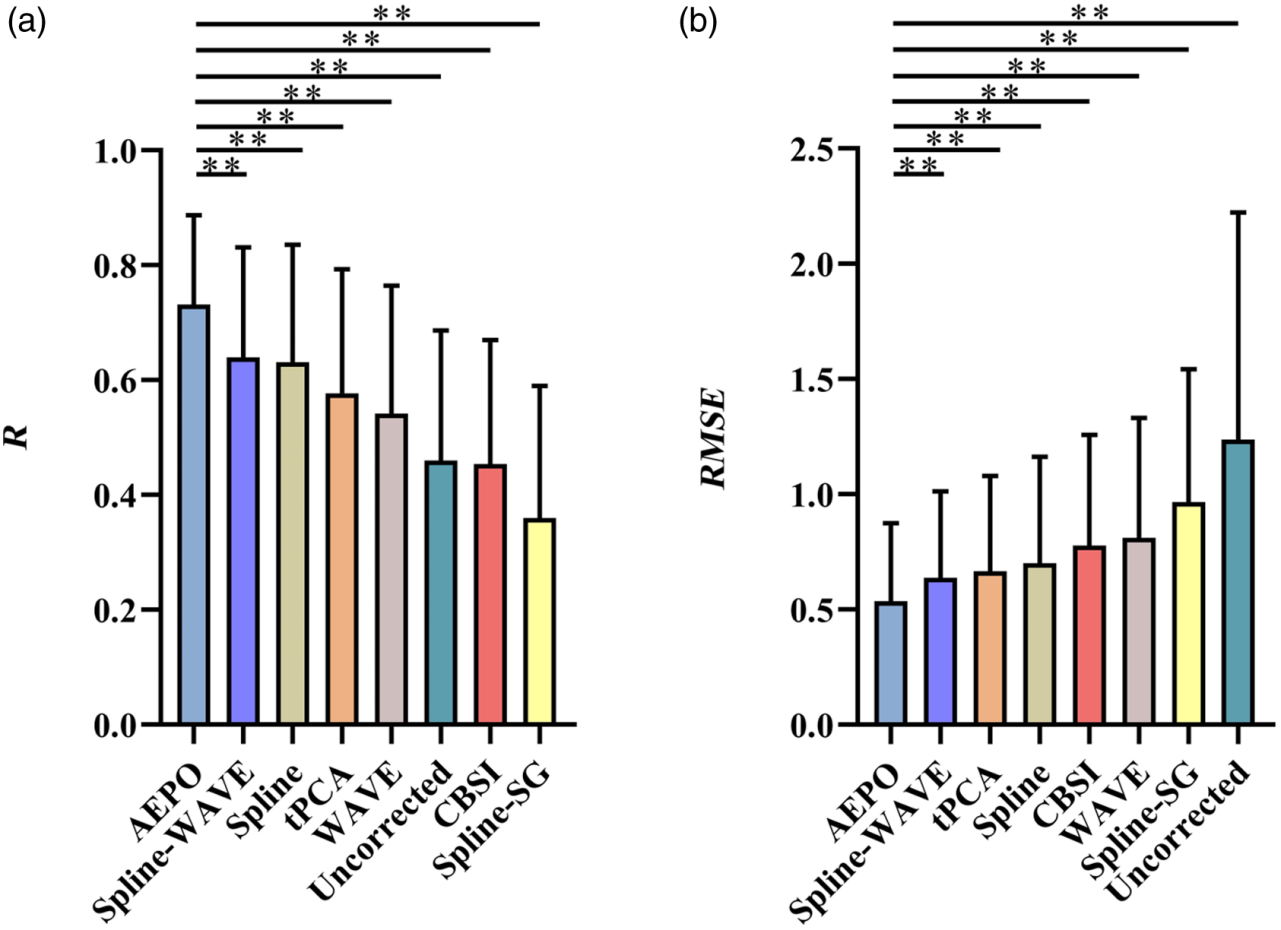
(a) R and (b) *RMSE* values for ΔHbO obtained by manual correction and different algorithms for the neonatal datasets.

Figure 8 shows the values of *R* and *RMSE* for the ability of different correction techniques to remove the artifacts in neonatal resting-state fNIRS data. Compared with the data with no correction artifacts (R=0.459±0.227, RMSE=1.237±0.986), CBSI (R=0.454±0.216, RMSE=0.777±0.480), and Spline-SG (R=0.360±0.230, RMSE=0.966±0.576) were inadequate in recovering the signals in neonatal data. Whereas AEPO (R=0.732±0.155, RMSE=0.536±0.339), WAVE (R=0.542±0.222, RMSE=0.811±0.520), tPCA (R=0.577±0.215, RMSE=0.666±0.413), Spline (R=0.631±0.205, RMSE=0.700±0.462), and Spline-WAVE (R=0.640±0.191, RMSE=0.637±0.375) alleviated the effects of motion artifacts on ΔHbO. Among the methods that showed improvements, the AEPO method performed better statistically in terms of *RMSE* and R (paired t-test, **p<0.01, Table 9) and produced amplitudes of ΔHbO that were closer to those of the manual correction method.

**Table 9:** P-values (paired t-tests) denoting improvement or significant improvement in R and *RMSE* using the method in the row over the method in the column for neonatal data.

R RMSE	Spline-WAVE	Spline-SG	WAVE	Spline	tPCA	CBSI	Uncorrected
**AEPO**	<0.01	<0.01	<0.01	<0.01	<0.01	<0.01	<0.01
	<0.01	<0.01	<0.01	<0.01	<0.01	<0.01	<0.01
**Spline-WAVE**	—	<0.01	<0.01	0.14	<0.01	<0.01	<0.01
	—	<0.01	<0.01	<0.01	<0.05	<0.01	<0.01
**Spline-SG**		—	<0.01	<0.01	<0.01	<0.01	<0.01
		—	<0.01	<0.01	<0.01	<0.01	<0.01
**WAVE**			—	<0.01	<0.01	<0.01	<0.01
			—	<0.01	<0.01	<0.05	<0.01
**Spline**				—	<0.01	<0.01	<0.01
				—	<0.05	<0.01	<0.01
**tPCA**					—	<0.01	<0.01
					—	<0.01	<0.01
**CBSI**						—	0.89
						—	<0.01

## 4 Discussion

In this study, we proposed a suitable denoising method for complex fNIRS signals in neonates. Typically, when collecting neonatal fNIRS data, motion artifacts contaminate the fNIRS signal owing to reasons such as head movements by the subject and detector not being firmly attached to the scalp. Motion artifacts of different sizes and frequencies are not easily and completely recognized by artifact detection procedures.^27^ In addition, the existing correction methods fail to consider the influence of the noise created by a mixture of multiple motion artifacts in neonatal fNIRS data on the recognition and correction algorithm, which reduces their ability to handle artifacts in neonatal data and correct them appropriately and adaptively.^27^ Therefore, our strategy was to first estimate the normal physiological oscillations in the neonatal signal such that motion artifacts of different amplitudes and frequencies could be identified to the maximum extent. After identifying the motion artifacts, we corrected the baseline shifts and spikes using Spline with Gaussian replacement to avoid residual artifacts. This study evaluated the performance of the proposed method using simulation data and real neonatal resting-state data. The performance was compared with that of commonly used artifact correction algorithms in the fNIRS field, which verified the advantages of our proposed method in preprocessing neonatal resting-state fNIRS data.

Based on the R and *RMSE* values, the CBSI method does not provide a significant advantage in the cases of simulated data and actual neonatal data (simulated data: R=0.578±0.113, RMSE=0.420±0.116; neonatal data: R=0.454±0.216, RMSE=0.777±0.480). According to our analysis, the changes in the HbO and HbR concentrations in head blood are not strictly negatively correlated in neonates in the resting-state.^23^^,^^50^ Moreover, in the study by Behrendt et al. on fNIRS of mother–infant interaction, HbO and HbR in infant cerebral blood were not strictly negatively correlated in the facial expression task.^10^ This is a characteristic of infant blood flow. However, this characteristic deviates from the assumptions of the CBSI method,^24^ causing variance between its corrected neonatal data and the manually corrected data.

Compared with the results without any correction processing (simulation data: R=0.645±0.109, RMSE=0.472±0.131; neonatal data: R=0.459±0.227, RMSE=1.237±0.986), the tPCA method failed to demonstrate improvements in the case of simulation data (R=0.613±0.117, RMSE=0.366±0.065), whereas in the case of neonatal data, a slight improvement in the correlation R was observed (R=0.577±0.215, RMSE=0.666±0.413). However, this improvement is usually not sufficient to meet the requirements for artifact removal, such as in neonatal brain network analysis. The tPCA method relies on the artifact recognition result.^27^ Second, it also requires that all artifacts be recognized as the main component.^23^ However, in actual neonatal data, artifacts with small amplitudes are not easily or completely recognized as the main component.^12^ In addition, tPCA directly removed the largest principal component of the proportion of variance in the data. However, these components may also contain changes in the blood flow.^51^ Owing to the above possible reasons, the effect of the tPCA correction was not obvious.

WAVE and Spline are effective in removing spike noise and baseline shift respectively from the neonatal data. However, they are not effective in handling serial disturbances in the neonatal data, caused by a combination of different proportions of spikes and baseline shifts. The differences between the approaches are influenced by the type of motion artifacts in the data and the detection effectiveness. Specifically, the WAVE approach was better at correcting for spike artifacts in the neonatal data (R=0.542±0.222, RMSE=0.811±0.520). However, when faced with noise consisting of spikes superimposed with baseline shifts, the WAVE method smears or smoothens this part of the artifact,^50^ leading to a greater degree of baseline shift. This problem persists even after changing different parameters. In contrast, Spline relies heavily on artifact detection. Factors such as inaccurate artifact detection and a higher amount of high-frequency noise in the neonatal data cause unstable results.^50^ Moreover, data processed with Spline usually contain residual spikes, especially at the sites of disturbance caused by noise (R=0.631±0.205, RMSE=0.700±0.462). In our study, the combination of Spline and WAVE produced slightly better results in the neonatal data than their individual results, with a reduced number of residual artifacts in the corrected signal. Similar conclusions were obtained in the study by Lorenzo et al., in which the combination of Spline and WAVE showed better performance on semi-simulated data, and recovered the majority of data affected by motion artifacts in all datasets.^27^ Therefore, we recommend the use of Spline-WAVE for complex fNIRS data rather than the Spline or wavelet method.

The Spline-SG method showed good recovery effect in the simulated data (R=0.828±0.078; RMSE=0.213±0.049). However, for actual neonatal data, the Spline-SG correction method performed poorly (R=0.360±0.230; RMSE=0.966±0.576). The reason for this result is that not all channels in the neonatal data used in this study had an SNR>3 (117 channels with SNR<3). Using SG filtering did not remove all artifacts, which is consistent with previous reports.^27^

The different motion correction methods showed varying performance for connectivity restoration. In the simulation data, AEPO, Spline-SG, WAVE, and Spline-WAVE recover better for the two types of significantly decreased connectivity. For AEPO, Spline-SG, and Spline-WAVE, the phenomenon of recovery is related to the modeling operations in the Spline.^38^ However all connectivity does not returned to its original level. For the WAVE method, when faced with continuous disturbance, the noiseless signal before and after disturbance does not change after correction. The residual baseline drift may be the reason for the connectivity not returning to its original state.^38^

The results of the combined simulation and neonatal data showed that the optimized method in this study had a better correction effect than the traditional algorithm (simulation data: R=0.860±0.037, RMSE=0.196±0.024, **p<0.01; neonatal data: R=0.732±0.155, RMSE=0.536±0.339, **p<0.01), while also displaying an improved effect on the restoration of connectivity in the simulation data. Because motion artifacts in neonatal data affect the calculation of the recognition thresholds, our strategy was to eliminate this effect using sliding windows and sorting. When processing neonatal fNIRS signals containing different motion artifacts, this strategy adaptively estimates the physiological oscillations in the noise-free part of the signal and then uses them as thresholds to maximize the objective recognition of artifacts. Thus, the threshold multiplier STDEVthresh has a smaller range. In addition, to solve the problem of residual artifacts, we used different treatments for different types of artifacts. Because Gaussian substitution can handle only spikes of a short duration, we recommend using Spline to remove baseline shifts. The signal is then passed to the Gaussian replacement algorithm to correct for high-frequency spikes. Thus, the Gaussian is not used to correct the baseline shift in the signal, which produces better results. Based on the results of our study, we recommend using the AEPO approach as a preprocessing method for neonatal fNIRS data. However, our method has some limitations. Only the resting-state fNIRS data of the neonates were processed in this study. In scenarios where the physiological oscillations have different magnitudes in the alternating task and resting states, our method may have over-correction problems. In the future, we will further optimize this aspect so that our method may be used as a preprocessing tool for physicians to monitor brain function in newborns.

## 5 Conclusion

Motion artifact correction is a fundamental preprocessing step for transforming raw fNIRS data into changes in the HbO concentration. Particularly, when the subjects are newborns or infants, the preprocessing results are critical for subsequent analysis. Traditional motion artifact correction faces certain challenges when processing data from such populations. This paper presented an artifact correction method suitable for neonatal data. The proposed method maximizes the identification of artifacts in the data based on the physiological oscillations in the signal and combines the advantages of both Spline and Gaussian replacement methods to provide a better correction effect for different types of noise in neonatal data. The application of the proposed method to simulated data and neonatal resting-state data showed that our method obtains faster and better results than other artifact correction methods. Hence, it is suitable for correcting artifacts in newborns data within short time, thereby improving the clinical applicability of the fNIRS technique to neonates.
